# Barriers among Danish women and general practitioners to raising the issue of intimate partner violence in general practice: a qualitative study

**DOI:** 10.1186/1472-6874-14-74

**Published:** 2014-06-03

**Authors:** Trine Mørk, Pernille Tanggaard Andersen, Ann Taket

**Affiliations:** 1 Institute of Marketing and Organisation, Aarhus University, Bartholins Allé 10, 8000 Aarhus C, Aarhus, Denmark; 2 Unit of Health Promotion Research, University of Southern Denmark, Niels Bohrs Vej 9, 6700 Esbjerg, Denmark; 3 School of Health and Social Development, Deakin University, Melbourne Burwood Campus, 221 Burwood Highway, Burwood, VIC 3125, Australia

**Keywords:** Intimate partner violence, Routine inquiry, General practitioners, Domestic violence, Social determinants of health, Partner violence

## Abstract

**Background:**

Thirty-five percent of Danish women experience sexual or physical violence in their lifetime. However, health care professionals are not in the practice of asking about intimate partner violence (IPV) in Denmark. It is currently unknown what hinders general practitioners from asking about partner violence and how Danish women would perceive such an inquiry. This aspect has not previously been explored in Denmark. An exploratory study was conducted to examine what hinders general practitioners (GPs) from asking and what Danish women’s views and attitudes are regarding being asked about IPV.

**Methods:**

Data were collected through individual and group interviews with a sample of three GPs and a diverse sample of 13 women, including both survivors of partner violence and those without any history of partner violence. An interpretative analysis was performed with the data.

**Results:**

This study provides important knowledge regarding the barriers and attitudes towards inquiry about IPV in primary care in Denmark. Results indicate that Denmark is facing the same challenges when responding to survivors of IPV as other similar countries, including Sweden, Norway, the UK, USA, and Australia. Danish women want general practitioners to ask about violence in a respectful and non-judgemental manner. However, general practitioners are resistant towards such an inquiry and would benefit from training regarding how to respond to women who have been exposed to IPV.

**Conclusions:**

It is acceptable to inquire about IPV with women in Denmark in a non-judgemental and respectful way. Informing about IPV prevalence is important prior to the inquiry. However, general practitioners require more awareness and training before a favourable environment for this change in procedure can be created. Further large-scale research is needed to support the evidence generated by this small study.

## Background

Every day the human rights of hundreds of thousands of women are violated all over the world. In many cases they are violated because of women’s unequal status in society [1]. Domestic violence is accepted across many layers of society and occurs among all socio-economic groups in all countries [2]. From a lifetime perspective, more than 30% of all women will experience violence and/or sexual violence [2,3]. The implications for women who experience intimate partner violence (IPV) are grave as it can lead to serious injury, disability, or death. Indirectly, IPV can lead to a number of health problems, such as a loss of personal autonomy, fertility challenges, substance abuse, depression, anxiety, and sleep and eating disorders [1-5]. It is estimated that IPV costs the Danish society a minimum of 280 million Danish Kroner per year [6]; however, this is likely to be grossly underestimated.

There are no relevant data from research or other investigations regarding inquiries about IPV in General Practice settings in Denmark. Thus, General Practitioners’ (GP) attitudes towards IPV and performing routine inquiries about it are still unknown. In general, the Danish healthcare system lacks information regarding IPV. Literature from other countries suggests that women want to be asked about violence in a safe, confidential, caring, and non-judgemental environment [7-9]. When professionals fail to address obvious signs of violence and the underlying causes of injuries, women do not feel that they are being respected. They feel unimportant, isolated, discouraged in their efforts to leave the relationship, and even more alienated from the rest of society [10]. Literature from other countries suggest that health professionals’ barriers towards routine inquiry are primarily, but not exclusively, about the lack of knowledge about prevalence within different groups of women, lack of experience in addressing IPV and lack of knowledge about specialised services that are available [11,12]. It is estimated that 28,000 Danish women each year experience IPV [6]. Of these 28,000 women, just 7% seek assistance at a shelter for abused women and their children [6]. These refuges offer a place to live for short or long periods and where women can receive help. The remaining 93% handle these situations by themselves or receive some assistance from different hotlines provided by NGOs such as the Mothers’ Help (Mødrehjælpen), which provides counselling, psychology sessions, and financial assistance [13]. Most women exposed to IPV never contact a refuge despite this being the only comprehensive service currently provided by the Danish welfare state.

Within the Danish system, general and private practices provide free medical assistance from a general practitioner. The general practitioners occupy a central position in the health service as they are the patients’ primary contact. General practitioners must ensure that patients are provided with proper treatment and referred to appropriate hospitals and specialists. There are approximately 4,100 general practitioners in Denmark who participate in the collective agreement with the public healthcare scheme. Each general practitioner has approximately 1,300 patients [14].

Survivors of IPV identify medical doctors as the healthcare professionals from whom they would most likely seek help [15]. Although it has not been considered in Denmark, routine inquiry and/or screening for IPV have been discussed and investigated for years in many neighbouring countries, such as Sweden, Norway, and the UK. Recent research from the UK shows promising results regarding a training and support programme that focuses on GPs and administrative staff. The results indicate an increase in referrals and disclosures [15].

The weave project from Australia was designed to evaluate whether a multi-faceted intervention in General Practice would increase women’s well-being, safety, and mental health. The intervention consisted of a screening with feedback, training for health providers, a brief counselling intervention for women who had experience of IPV, and minimum organisational change [16]. An evaluation of this intervention provided some promising results [17].

The current paper presents the results of an exploratory investigation in Denmark. The views of three different respondent groups were examined, as follows: general practitioners, survivors of IPV, and women who did not report any experiences with IPV. These groups can be expected to have different levels of acceptance and attitudes towards introducing inquiry about intimate partner violence in the General Practice setting. The research question for this study was the following: *What attitudes and barriers exist among General Practitioners, survivors of partner violence, and women with no history of violence towards routine inquiry*^a^*in General Practice?*

## Methods

### Study design

This was an exploratory qualitative study using individual and group interviews with GPs, survivors of IPV, and women with no history of violence. These respondent groups were selected given that they represent three different groups that would be directly affected by any procedural change regarding an inquiry about IPV in General Practice. In general, this study is based on an interpretative scientific approach. Elements from grounded theory [18] were used to analyse the qualitative data that were generated. This is discussed in detail in the section regarding analysis and coding.

### Ethical considerations

The field of IPV research raises important ethical and methodological safety issues and confidentiality issues. The interviewer’s training and skills are important. In this study, the guidelines for research on domestic violence against women, as outlined by the Department of Gender and Woman’s Health at WHO, were followed to insure that no respondents or researchers were at risk [19]. In Denmark, approval is not necessary from an ethics committee when human tissue is not involved in the study. Regardless, the current research was conducted following standard research ethics guidelines [19] and all participants provided oral informed consent. Data were collected in April and May of 2012.

### Description of recruitment of interviewees

The three respondent groups were recruited using different forms of convenience sampling. This was necessary given the timescale available for the current research, which was conducted to fulfil the requirements of a Master’s degree.

It was not possible to mail research invitations to all of the GPs in practice in the lead author’s local area (East Jutland); therefore, GPs were recruited using the snowball method. Email invitations were sent to two GPs in the researcher’s network with a request that they send the invitations to their colleagues. It is not possible to know the total number of GPs who received that email. All GPs in practice were eligible and all who responded were interviewed. GPs were offered an individual interview at a time and date of their choosing.

Recruitment for survivors of IPV was through two Danish refuges in East Jutland. The directors of the refuges posted a flyer inviting survivors of IPV to a group interview at the refuge. Eligible participants were all women at the refuge who spoke Danish. No pressure to attend the interviews was placed on the women living at the refuges.

A convenience sample of women with no history of violence was recruited through Facebook. Eligible women were over the age of 18 years, able to speak Danish, had no history of violence or sexual assault, and were able to attend the single group interview that was offered for this group of respondents.

### Interviews

The first author conducted all of the interviews, which were partly semi-structured and partly open. All interviews were based on a thematic interview guide. Given that there were three different respondent groups in this study, three guides were developed and used. The guides were inspired by a Swedish research project from 2002 called, “Tack for att ni frågar” (“Thanks for asking”), which was initiated by the Swedish National Board of Social Services [20]. This project was similar to the current study with regard to their subjects and methods, and similar to the questions in the Partner Violence Screen [21]. Effort was made to operationalize the research question into the interview questions. As the interview questions in this current study were used to approach the research question from several different angles, the information and perspectives that were obtained regarding the subject were very rich. The questions were presented in a concise manner with an emphasis on questions that encouraged participants to reflect and, in group interviews, to discuss amongst themselves. Examples of areas covered in the three interview guides are shown in Table 1. To avoid participants feeling that there were right or wrong answers, emphasis was placed on questions starting with “How” and “What” and explorative questions. These kinds of questions encourage participants to provide spontaneous descriptions of the phenomenon that is being investigated.

**Table 1:** Examples of areas covered in the interview guide

**Category of respondent**	**Areas covered**
GPs	Do you believe IPV is a problem in Denmark? Why/why not?
	What is your attitude towards routinely asking about domestic violence?
	When do you ask your patients if they have been exposed to IPV?
	What would encourage you to ask more women about IPV?
Survivors of IPV	How would you react if your GP asked you questions regarding IPV?
	Can you think of any drawbacks to being asked about IPV by your GP? If so, what are they?
	What response would you want from health professionals regarding the violence that you and your children have experienced?
	How do you think we can help women around the country get out of violent relationships?
Women who have not experienced violence	Do you think it should be the GP’s job to ask all women about their relationships with their husbands or boyfriends?
	How would you react if your GP asked you about IPV?
	Can you think of any drawbacks to being asked about IPV by your GP? If so, what are they?
	How do you think we can help women get out of violent relationships?

Interviews were conducted based on Kvale’s procedures [22]. During the group interviews, an observer was present to monitor the processing and body language of the participants. The length of the interviews varied from 25 minutes to 1 hour and 50 minutes, with the GP interview being the ones lasting approximately 30 minutes. All interviews were recorded digitally (with the interviewees’ permission) and transcribed verbatim by the interviewer (i.e., the first author).

### Coding and analysis

The interviewer (i.e., the first author) coded the interviews in multiple rounds. The first stage of the analysis applied open coding [23], and the results of this coding were discussed with the second author. Following this, the first author designed a coding scheme, and then the second author revised this scheme and suggested new concepts. After the application of this coding scheme, analytic themes were generated and selective coding was used to explore these themes in more detail. Parent categories were derived to capture the themes that dominated participants’ responses to the questions. Throughout this stage, coding schemes and analytical themes were discussed and revised accordingly by the first two authors. After identifying the parent categories, translation from Danish to English occurred. Then, the third author provided comments and suggestions for refinements to the parent categories. These categories were explored in the final stage of the analysis.

## Results

Recruitment yielded 3 GPs, 8 women who were survivors of IPV, and 5 women with no history of IPV. Table 2 presents a summary of the characteristics for the three groups of research participants. The three GPs who were interviewed had their practices in different settings, with one in an urban setting and two in smaller towns. Each of the GPs had a patient group that included a variety of income levels. One woman who was a survivor of IPV was not recruited at a refuge; rather, she contacted the researcher in response to an invitation posted on Facebook to recruit women with no history of IPV. Given that the group interviews at the refuges had already been conducted, this woman was offered an individual interview, which was an opportunistic form of recruitment.

**Table 2:** Characteristics of the research participants by respondent group

**Group**	**Survivors of intimate partner violence (n =8)**	**Women who have not experienced violence (n = 5)**	**General practitioners (n = 3)**
Age group	18-25: 3	18-25: 1	18-25: 0
	26-50: 4	26-50: 4	26-50: 2
	51-74: 1	51-74: 0	51-74: 1
Mean age	36	36.6	50.6
Number of children	None: 3	None: 3	No data
	One: 2	One: 0	
	Two: 3	Two: 2	
Employment status	Employed 1	Employed 4	Employed 3
	Unemployed 6	Unemployed 1	
	Retired 1		
Ethnicity	Danish 3	Danish 5	Danish 3
	Other Scandinavian 1		
	Other ethnicity 4		

Analyses revealed that the three different groups of respondents had similar concerns, with some variation according to their group’s particular perspective. The GPs considered time and cost/benefit issues and discussed the awkwardness of confronting a woman with such a personal question. The survivors of IPV demonstrated great confidence in their GPs while simultaneously worrying about breaches in confidentiality. The women with no experience of violence demanded more knowledge on the subject and thought that questions regarding violence should be put into context in a consultation. The next sections present the attitudes, perspectives, and opinions expressed by each respondent group regarding the barriers to implementing routine inquiries. In the following, all of the names are pseudonyms.

### GPs - views and perspectives

The main issue for the GPs was the awkwardness of discussing IPV with women. Although they did not overly emphasise the time issue, it was of some significance as most GPs have between 5 to 15 minutes per patient to cover the issues that need to be addressed during the consultation. When GPs considered the time issue, they were more concerned that IPV screening/routine inquiry would be too large a burden on the consultation and that this screening was not related to their competencies. None of the GPs who were interviewed had much experience with IPV. Additionally, in their opinion, IPV did not occur much in their practices:

“I think you should find the right formulations. You should have learned about it so it seems natural if you are to ask about it routinely. Because, in the majority of the consultations that we have here with women, it will not be relevant … I have always been fortunate enough to work in places where that kind of problem did not occur” (GP, 6 years in private practice).

Cost and benefit issues were raised both for and against screening. The GPs interviewed shared the view that the economic costs were too high compared to how many women they believed were affected by IPV. Some perceived a flexible form of routine inquiry as an extra pressure given that it relies more on their interpersonal skills. This sample of GPs thus did not deem it reasonable to introduce routine inquiry in private practice. They insisted that they should not ask every woman during consultations. The GPs had a high degree of confidence in their own abilities to detect abused women.

When the GPs were interviewed about IPV, all of them stated that they considered this to be a serious issue. All responded that they ask about bruises that do not match individuals’ explanations, just not in a direct manner. During this type of situation, age and experience played a role, as all of the GPs mentioned that asking became less difficult with age and experience. However, age and experience did not influence the GPs’ perceptions of their ability to detect IPV, as they all reported that they felt very comfortable inquiring about IPV on suspicion and did not hesitate to respond that they recognised the signs of IPV. Their statements that they asked patients when they recognised signs of IPV are difficult to reconcile with the self-reported number of times they asked (see Table 3), given that 35% of Danish women are exposed to violence or sexual assaults in their lifetime. It is important to note that all of the GPs spoke almost exclusively about the physical signs of IPV.

**Table 3:** Approaching IPV in private practice

**GP**	**Years in private practice**	**Estimated total number of inquiries about IPV**
Female GP	6	Less than 5/less than once a year
Male GP 1	15	approx. 20/approx. 1.3 times a year
Male GP 2	28	approx. 100 /approx. 3.6 times a year

One GP critiqued the judicial system and the response system when women disclose abuse, arguing that the response should cover the ‘bigger picture’ to provide more coherence. The GP criticised the judicial system for being slow with regard to divorce, separating the woman from her perpetrator, and providing her with financial freedom.

### Survivors of IPV – views and perspectives

A number of the survivors of IPV initially stated that they did not want their GPs to ask them about IPV unless they came to the clinic with bruises caused by IPV or looked sad.

“In some ways, I would find it offensive if a GP asked me about that. Of course, if a patient comes to the clinic and starts crying, then he can ask. But if I came with bruises and I had not been exposed to IPV and he kept asking, then I guess I would shut off and say, “What do you mean by that?” But, on the other hand, if a woman came in with internal bleeding due to being punched, then I think he should 100% ask and interfere because that’s not something you can get in some other way, in my opinion at least” (Aisha, no children).

This statement indicates that the GPs should guess when a bruise, bleed or mental condition is due to IPV occurring at home. In general, the survivors of IPV trusted that their GP could see what was going on, as the women expected the doctors to know whether they were sad or struggling at home with serious issues.

During the course of the interviews, this attitude became less dominant and the respondents focused more on accepting the inquiry provided it was not presented in an impersonal manner: “I don’t just want to be an X on a piece of paper”. Thus, the GPs’ empathy, knowledge, and willingness to address the issue were important factors during consultations regarding violence. In addition, the coherence of the response system, (responses from police, the judicial system, the help provided by the refuges, and the support from the municipality and social workers), was regarded as insufficient and full of gaps by all of the participants. The participants mention poor communication between police and refuge and GP and refuge, poor security at the refuges, where the women are urged to take care of their daily life as normal, paying bills and such, risking bumping into their perpetrator.

During the interviews, all of the women with experience of IPV agreed that they would not mind questions about IPV if the GPs asked in an empathic, sensitive, and non-judgemental manner. Most of the women who had IPV experience (6 of 8) were very firm in their responses to whether the GPs should ask about IPV. They wanted to be asked because they wanted help and they did not feel that they were able to ask for help themselves:

“I want him to ask me how I am doing, because you can’t say it yourself without him asking about how things are going at home. Because you don’t know how to say it at all. I would have told him if he had asked. It would have saved me from 12 years” (Amina, no children).

This quote suggests that when no one listens or asks about the violence occurring in women’s lives, the women need to “pull it together” day after day. Therefore, they are most likely discouraged from leaving the relationships when no one addresses the issue of violence.

“Certainly the GP should ask. It depends a little on the context… If my GP had asked me directly, I would have gotten help earlier from Mothers’ Help and maybe it would not have escalated so much… The longer you are dragged through this, being exposed to violence… It’s such an extreme experience that, from health and societal perspectives, the sooner that people receive help, the better” (Charlotte, one child).

This quote suggests that GPs should act on their suspicions; however, it can be difficult for GPs to act when they experience immense time pressures. The abused women often stated that GPs provide patients with the impression that they are very busy. The women felt that this created an unsupportive environment where patients were not likely to confide in their GPs. They also stated that a generic form of questioning was not acceptable, as most of the women emphasised a personal approach. Thus, abused women preferred a more flexible routine inquiry to a formalised screening procedure. Routine inquiry provides the GPs with the opportunity to customise the questions to specific situations. The survivors of IPV emphasised the importance of medical doctors being trained in the aetiology of violence, its prevalence, and inquiry techniques.

There were a number of contradictions evident in the views of the survivors of IPV. On one hand, they believed in the GPs’ ability to perceive the abuse that they were hiding. On the other hand, they discussed how dangerous it could be if the GPs did not fully understand the implications of harmful advice and breaches of confidentiality for women living in violent relationships.

“The GPs should be careful about how they counsel their patients, for instance, the idea of just leaving him, that’s not so easy. If it was easy, you would have done it a long time ago” (Aisha, no children).

Although they knew that GPs were bound by confidentiality, the women were concerned about the confidentiality issue.

“My big brother got some information from my GP. It is about honour. It can be very dangerous to discuss this in some cultures. So, the doctor thinks, well, it is her own brother, he will probably help her. I have experienced that once and that’s a drawback” (Amina, no children).

Although confidentiality issues were discussed, the abused women thought that General Practice was the right forum. Based on the women’s concerns, providing further education and a proper referral system may eliminate a number of issues, allowing routine inquiry to be a positive experience.

### Women with no experience of violence – views and perspectives

When discussing routine inquiry with the women with no experience of violence, these women believed that the GPs recognised the signs of abuse and asked the patients about it. The majority of the women who participated in this study (4 of 5) stated that it was acceptable to ask questions about violence during clinical visits when presented in a non-judgemental and empathic way. Their statements reflected that knowledge about the subject was important. The greater their knowledge on the subject, the more the women accepted routine inquiry.

“It gives me an impression of the doctor as being more holistic, if you ask about that kind of thing, like the doctor is more sensitive. I think it could have a positive effect, given that it’s a topic to be addressed with your own GP, because in time these kinds of things will become less taboo. Because it’s something that you are asked about by your GP, ergo it’s something we talk about. In Denmark, we also talk about if we are exposed to violence” (Rikke, no children).

All of the women who had not been exposed to violence emphasised the importance of providing information in some kind of form prior to any questioning so that the context was natural. During the group interview, there was significant discussion about how to ask about abuse appropriately and in what context it should be asked about. The women agreed that inquiry was a good idea if the context was appropriate and the GP was empathic.

One woman was reluctant to accept being asked routinely about violence. This suggests that there are women who may feel caught off guard and insulted if they are not appropriately informed about IPV prior to an inquiry. Although only a small percentage of women opposed being questioned about IPV, it is important to consider their objections in any future interventions.

## Discussion

The results regarding the acceptability of GPs inquiring about IPV in an empathic, sensitive, and non-judgemental manner are comparable to those from many investigations conducted in other countries (for example [7,24-27]) and indicate that response systems to IPV used in other countries e.g. the UK, Australia, USA, and Sweden could be transferred into the Danish context . The importance of understanding the prevalence of IPV in Denmark is necessary as well as a positive and trusting relationship between women and their GPs.

### GPs – practices and professionalism

The medical doctors that participated in this study lack training in communication and, in general, lack knowledge about domestic violence. Although there has been a change in culture from the traditional authoritarian treatment provider to the more patient-centred and dialogue-oriented approach in Denmark [28], there will always be a power imbalance between GPs and patients. This power imbalance means that sensitive topics can be difficult to address during consultations.

### A gap between prevalence and inquiry

The GPs in the study reported that they inquired about abuse when they had a good reason. However, the self-reported number of inquiries did not correspond with the number of women affected by violence [29]. One issue with IPV is that even after being shown IPV statistics at the beginning of the interview, the GPs’ interviews revealed that they reflect society’s view on the prevalence and socio-economic factors that influence IPV. They thus stated that routine inquiry about IPV was unnecessary and an expensive extra burden for them with little relevance for private practice.

### Awkwardness of inquiry

The GPs in the study were preoccupied with the violation of their patients’ privacy. Time and economic issues were mentioned as important challenges within the practice, although the emphasis was primarily placed on the awkwardness of the inquiry. These challenges are important factors to include when designing an intervention and to consider when determining the consequences of a possible disclosure, as in: Is there an appropriate response system in place? Feder et al. [15] showed that training health care professionals more than doubled the amount of disclosures in private practice. Inquiries by trained health professionals can help the survivors of IPV to move on with their lives and may do more good than harm [15]. However, in the public sphere, there seems to be a very high degree of respect for privacy to the extent that medical doctors hesitate to address obvious signs of abuse. This indicates that IPV is perceived as a personal issue that should not be interfered with unless the abused women ask for help.

### Improving the system

Currently, individual GPs decide how they prefer to approach IPV, if they do anything at all. Some materials have been produced for healthcare professionals to use [30], but it is up to the individual GPs to provide that information at the clinic. In general, GPs, emergency rooms, midwives, and other healthcare professionals only (but not always) address IPV when there are physical signs that cannot be explained by an accident. According to most of the women and GPs in this study (13 of 16), the referral system could be improved. The GPs only had very superficial knowledge about the possibilities for referrals. All of the GPs knew about women’s refuges, yet none of them mentioned The Mothers’ Help, where abused women are provided with counselling, therapy, and financial aid [13].

The women in this study believed that GPs knew when and how to ask difficult questions, and they agreed that it was of great importance that they were asked about IPV in a sensitive manner. The survivors of IPV reported that they wanted more help from the police, GPs, social workers, psychologists, and refuges and that they wanted more attention directed toward the subject of IPV in general. Survivors wanted more coherence from the response system, given that they had difficulties disclosing abuse. The women had good ideas about how the response system should be designed and showed a mistaken trust in the GPs’ ability to perceive their troubles through their façades.

### Sensitivity in inquiry

In a study by Boyle and Jones [31], 8.4% of women believed that inquiry was unacceptable, yet this relatively high percentage may be due to the phrasing of the question, as follows: “Have you ever been hit, kicked, pushed, slapped or in any other way harmed by a current partner or ex-partner within the last 12 months?” The women in the current study were asked how they would feel about being asked in that manner and they found it unacceptable, stating that it was almost like they were being hit by the words. This question was derived from the “Revised Conflict Tactics Scale”, which is a validated and frequently used screening tool [32]. A study by Morse [27] showed that women were concerned about advice regarding leaving the perpetrator. In Morse’s study, 72% of GPs gave women the impression that they should quickly leave their partners [27]. Often family situations and the support provided to women and children make the process of leaving their perpetrators more complex; therefore, providing advice with little to no knowledge of the aetiology of violence can be fatal for women. The GPs need to take the issue of security into account when discussing options for help with abused women. The interviews revealed that there are also external implications evident apart from private practices, such as judicial issues.

### Security and confidentiality

Survivors of IPV wanted to have a system in which people were not afraid to discuss IPV. They asked for coherence in the response system such that GPs who inquired about IPV could provide referrals and services, including options for urgent access. Survivors were preoccupied with security and thought that the police should be able to help them more when called to their home. Thus, women in this study generally wanted to be asked questions about IPV by health professionals in a non-judgemental manner. They wanted the option for help if necessary. They stated that they felt insecure at the refuges and that they needed more help when interacting with the municipality due to social workers’ lack of understanding with regard to their situation and sometimes the presence of harsh rhetoric.

### Contextualising the results

There are a number of underlying factors that are relevant to understanding why the issue is approached in the manner that it currently is. In general, Danish society is constructed so that considerations for abused women and children have not been prioritised with regard to the prevalence and severe impact that IPV has on their health and personal autonomy. Privacy is a key word in this analysis: IPV is still treated in a manner that supports the reaction that it is a purely private matter when a man is abusing his partner.

There is a general lack of knowledge regarding the normalisation process, which is how abuse slowly becomes the new normal as abused individuals become increasingly dependent on their abusers. This dependence is due to the constant shift between kindness and abuse by the abusers and is a product of the gender inequality evident in Denmark and around the world. The findings in the current qualitative Danish study are consistent with research conducted in Sweden, the UK, and USA [24-27,33,34].

These results reveal that there is a gap between the survivors of IPV and GPs regarding the GPs’ considerations for the women’s feelings and the women’s desire to be asked directly about abuse. There is also a gap between the seriousness of the problem and the response system that is in place (e.g., the police, the judicial system, and the refuges). Although all of the groups recognised that women living with IPV required help, the GPs and the women with no history of violence were sceptical about making any inquiries on a routine basis.

Given abused women’s reluctance to disclose and the GPs erroneous confidence in their own abilities to detect abused women, very few women are provided with the help needed to leave a violent relationship or appropriate counselling. The GPs should provide expert information and treatment that are directed at the cause of the symptoms of the patient. If they overlook the underlying reasons for various illnesses, the GPs may fail to treat the patient as a whole person and fail to address the actual cause of the patient’s problems.

Although Denmark is one of the more gender equal countries in the world [35], the lifetime prevalence for experiencing violence and/or sexual violence is equivalent to the worldwide average of 33% (Denmark: 35%) [29]. The consequences of not detecting abused women and affected children are evident in the number of lives lost and quality of life lost, as well as in the huge economic burden that abuse places on society. Internationally, there is an increasing amount of research within General Practice that is focused on designing appropriate interventions to reduce IPV and its consequences for both women and children.

### Limitations of the current study

There are several limitations to the current study. First, all of the women were asked to discuss a hypothetical routine inquiry situation, which may reflect a greater degree of reluctance in terms of acceptance, given that studies show that women who have experienced routine inquiry are more likely to favour it [36]. The GPs were asked to account for the number of times that they had inquired about IPV in their private practices, which could reflect recall-bias and over-reporting. The number of respondents included in this study could have been higher. The number of GPs was particularly small. Interviewing more GPs would have been preferable, however, due to time constraints, this was not possible. However, it is reassuring that, despite differences in ages and genders, the GPs’ responses were very similar. Furthermore, their responses corresponded with a number of findings from studies elsewhere. Other limitations include that the respondents could have been recruited from different geographical areas in Denmark and the groups of women could have been matched to a greater degree. The small sample size means that the external validity of the current study is limited.

Despite limitations in the size of the study population and the possible lack of exhaustive responses, the analytical conclusions may indicate that the result of this study are generalizable, particularly given that the results for the attitudes and barriers in the current study were consistent with relevant literature from similar countries. Thus, this study supports conducting more exhaustive investigations regarding the issue of IPV in Denmark.

## Conclusions

The overall conclusion of the study is that there are differences in attitudes regarding IPV among GPs, survivors of IPV, and women with no history of violence. The GPs believe that they are able to recognize victims of IPV and do not believe that routine inquiry is a useful or appropriate tool for their private practice. The GPs have poor skills with regard to detecting IPV and lack knowledge of the consequences of IPV. They believe that the normal practice of seeing a patient is sufficient for detecting that a woman is being abused. Women believe that routine inquiry is acceptable when general information is provided first and the inquiry is conducted in a non-judgemental way. Emphasis is placed on confidentiality, coherence, security, and respect for the women as real people. The women preferred to be asked, as they do not know how to raise the issue themselves. In sum, the survivors of IPV are reluctant to disclose abuse and the GPs falsely believe that they are able to detect abuse. This results in very few women receiving help to leave abusive relationships.

It is important to note that the attitudes of Danish GPs have never been investigated before; therefore, this area of research is important given the amount of information needed before real steps can be taken towards designing a comprehensive framework for the early detection of IPV. Society should not under-recognize the value of being “seen”, even when women remain within abusive relationships and are subjected to further abuse. This point was emphasised by the participants in the current study.

Findings from this study indicate that Denmark does not differ from other similar countries with regard to IPV; therefore, successful interventions from the UK, Australia, USA, and Sweden could be successful in a Danish setting. Future research should investigate how a possible framework for the early detection of IPV might be implemented in Denmark with private practice as the platform for its implementation.

There appears to be a general reluctance to address the issue of IPV akin to a fear of ‘opening a can of worms’. This study emphasises the importance of applying a bolder rhetoric to send the message to all stakeholders dealing with IPV that it is not a private matter, it is not accepted nor tolerated in society, and all means will be utilised to end abuse against women. One billion women will be raped or beaten in their lifetime, which is one-third of the world’s female population. This is not only a woman’s issue; this is a global and local public health crisis.

## Endnote

^a^Routine inquiry in this context refers to “asking all people within certain parameters about the experience of domestic violence/IPV, regardless of whether there are any signs of abuse or domestic violence/IPV is suspected” [33]. The emphasis was on asking all women in an appropriate way and providing them with information regarding possibilities for help and basic knowledge on the subject.

## Abbreviations

GP: General practitioner; IPV: Intimate partner violence.

## Competing interests

The authors declare that they have no competing interests.

## Authors’ contributions

TM conducted the current study as a part of her Master’s degree and produced the first draft of the paper. PTA was the main supervisor and AT was the co-supervisor for this study. All authors contributed to the writing of this paper. All authors read and approved the final manuscript.

## Authors’ information

The work described in this paper was conducted to fulfil the requirement of a Master’s degree at the University of Southern Denmark.

## Pre-publication history

The pre-publication history for this paper can be accessed here:

http://www.biomedcentral.com/1472-6874/14/74/prepub
